# Increased Carotid Intima-Media Thickness in Asymptomatic Individuals Is Associated with the *PCSK9* (rs2149041) Gene Polymorphism in the Mexican Mestizo Population: Results of the GEA Cohort

**DOI:** 10.3390/life12101531

**Published:** 2022-09-30

**Authors:** Rosalinda Posadas-Sánchez, Gilberto Vargas-Alarcón, Óscar Pérez-Méndez, Nonanzit Pérez-Hernández, José Manuel Rodríguez-Pérez

**Affiliations:** 1 Departamento de Endocrinología, Instituto Nacional de Cardiología Ignacio Chávez, Ciudad de México 14080, Mexico; 2 Departamento de Biología Molecular, Instituto Nacional de Cardiología Ignacio Chávez, Ciudad de México 14080, Mexico

**Keywords:** carotid intima-media thickness, *PCSK9* gene, healthy individuals, coronary artery calcium, case-control study, single nucleotide polymorphism

## Abstract

The increase in carotid intima-media thickness (CIMT) and coronary artery calcification (CAC) are features of subclinical atherosclerosis that might be determined by the genetic background of patients. Among the multiple risk factors, the proprotein convertase subtilisin kexin type 9 (PCSK9) has a great impact on atheroma development. Then, we focused on the potential association of the *PCSK9* gene polymorphism (rs2149041) with the risk of an increased CIMT. We included 881 unrelated, asymptomatic individuals (732 normal CIMT and 149 increased CIMT) who lacked coronary calcification (CAC score = 0). Under the recessive inheritance model and adjusted by several cardiovascular risk factors, the rs2149041 polymorphism, determined by TaqMan genotyping assay, was associated with a high risk of increased CIMT (OR = 2.10, 95% IC = 1.26–3.47, P recessive = 0.004). Our results suggest that the rs2149041 polymorphism could be a risk marker for increased CIMT in asymptomatic individuals without coronary artery disease determined by the absence of a CAC score.

## 1. Introduction

Atherosclerosis is a multifactorial pathology usually leading to blood vessel stenosis, it involves chronic damage to artery walls caused by inflammation, lipid metabolism abnormalities and thrombosis dysregulation, among other [1,2,3,4,5].

An early identification of atherosclerosis intermediate phenotype traits is essential to prevent future clinical events, including the evaluation of genetic risk factors in different populations [6]. In this regard, an increased thickness of the intima and media layers of the carotid artery (carotid intima-media thickness or CIMT) is an important indicator of arterial remodeling that suggests an intermediate phenotype of atherosclerosis. Furthermore, CIMT is considered a non-invasive marker of subclinical atherosclerosis (SA) and cardiovascular disease (CVD) [7,8,9,10,11,12,13,14]. 

Previous reports have shown a heritability trait of CIMT [15,16,17] indicating that its genetic determinants may be useful to early detect pathological processes. In this regard, Willeit et al. reported an interesting large meta-analysis, included 119 clinical trials and involved 100,667 patients to assess the impact of the reduction in cardiovascular disease risk associated with the reduction of CIMT progression by therapeutic intervention. They also evaluated the association on CIMT progression on cardiovascular disease risk and research, and intervention [18].

In this context, the proprotein convertase subtilisin kexin type 9 (PCSK9) is a serine protease with a key role in regulating the low-density lipoproteins (LDL)-cholesterol homeostasis. PCSK9 is synthesized mainly in the liver, where it contributes to hepatic LDL receptor (LDL-R) degradation [19,20]. Therefore, PCSK9 decreases the hepatic clearance of LDL, leading to increased LDL-cholesterol plasma levels and a higher risk of atherosclerosis [21].

Additional studies have informed results with respect to the association between PCSK9 circulating levels and CIMT. Nevertheless, these data have been contradictory, with negative and positive associations, for instance, an association was reported in hypercholesterolemia familiar [22], subclinical carotid atherosclerosis [23], also in the progression of carotid atherosclerosis [24], whereas no association was observed with measures of vascular health [25], subclinical atherosclerosis of extracranial carotid arteries [26].

The human *PCSK9* gene is located on chromosome 1p32.3; it contains 12 exons that encode a 692 amino acid glycoprotein. This gene has different polymorphic sites that have been associated with the development of coronary artery disease (CAD) and other vascular disorders [27,28,29,30]. On the other hand, the possible association between the *PCSK9* (rs2149041) gene polymorphism and CIMT in asymptomatic individuals has not been described. However, there are few studies related to other *PCSK9* gene polymorphisms and CIMT. In this regard, an interesting study performed in the STANISLAS cohort (longitudinal familial cohort from the Lorraine region of France) reported that the polymorphism (rs562556) and increased PCSK9 levels were associated with the presence of arterial plaques in a healthy population; whereas no significant association between the rs562556 and CIMT was found [31]. Another report by Norata et al. found the association of PCSK9 (rs505151) polymorphism with high CIMT progression in the general population belongs to the PLIC study [20]. Therefore, our aim was to explore whether there is an association of the *PCSK9* (rs2149041) gene polymorphism with the risk of developing increased CIMT in asymptomatic individuals Mexican mestizo individuals. 

## 2. Materials and Methods

### 2.1. Design and Participants

This is a cross-sectional study, nested in the Genetics of Atherosclerotic Disease (GEA) study. The GEA cohort was designed at the Instituto Nacional de Cardiología Ignacio Chávez (INCICh) to establish the genetic basis that may be associated with the incidence of CAD, traditional risk factors, and with recently described risk factors of the disease [32]; particularly for the present report, we included the basal measurements obtained at the moment of recruitment.

**Figure 1:**
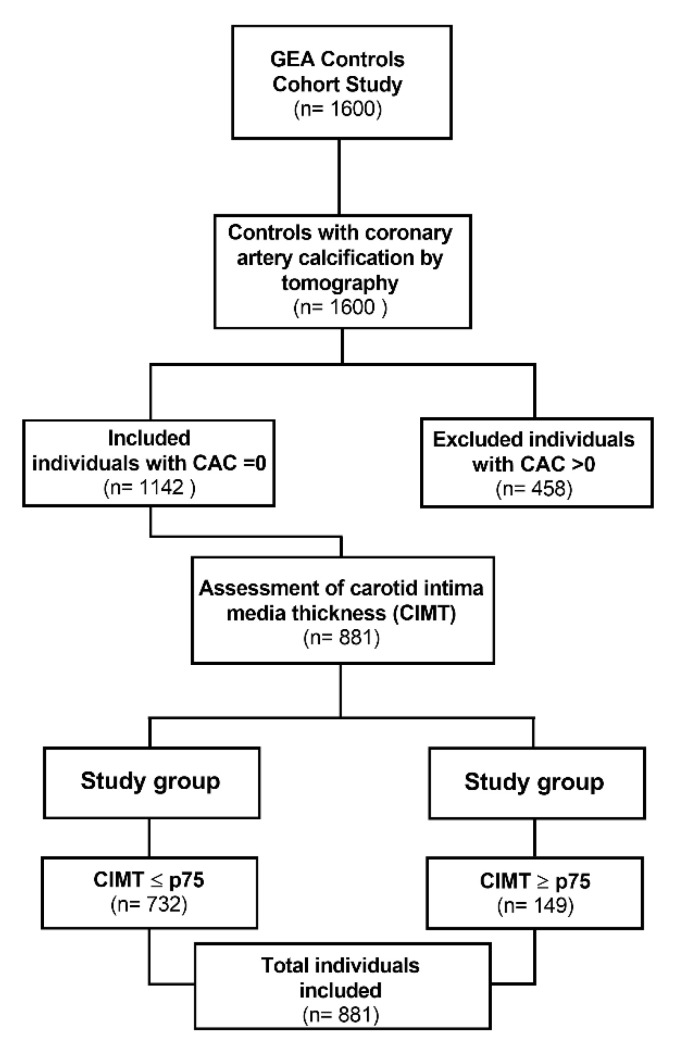
Flowchart for study selection.

The present study included 881 unrelated individuals whose coronary calcium score was 0, and who had no personal or family history of CAD or other cardiovascular diseases (Figure 1). All participants self-reported a Mexican mestizo ancestry (of at least three generations). These participants were chosen from donors attending the blood bank or were enrolled through social services or primary care centers from 2008 to 2013 in our institution (INCICh). Consecutive blood donors or non-consanguineal relatives of patients were invited to the GEA cohort. Once recruited, their medical histories, anthropometry, and demographic data were registered. Medical exploration, chest, and abdomen computed tomography, CIMT, biochemical analyses were performed. Individuals with a coronary calcium score = 0, calculated as described below. The study was conducted following the guidelines of the Declaration of Helsinki and approved by the Research and Ethics Committee of The Instituto Nacional de Cardiología Ignacio Chávez (protocol code 15-915). Also, informed consent was obtained from all subjects involved in the study.

### 2.2. Biochemical Determinations

Peripheral blood samples were collected from each participant after a 12 h fast. Biochemical measurements were determined in fresh samples using standardized protocols, and were processed with enzymatic colorimetric procedures in a Hitachi model 902 autoanalyzer (Hitachi LTD, Tokyo, Japan). Body mass index (BMI) was calculated as weight (kg) divided by squared height (m^2^); LDL-C was assessed according to the DeLong and Friedewald methods [33], whereas non-high-density lipoprotein cholesterol (non-HDL-C) was calculated by subtracting HDL-C from total cholesterol (TC) plasma concentrations. 

Adipose tissue insulin resistance (Adipo-IR) index was estimated as the product of fasting plasma free fatty acids (FFA) and insulin concentration as follows: Adipo-IR index (Adipo-IR = FFA [mmol/L] × insulin concentration [µIU/L] [34].

Type 2 diabetes mellitus (T2DM) was defined according to the American Diabetes Association criteria, with fasting glucose plasma levels > 125 mg/dL or when individuals reported medical prescription of hypoglycemic agents or they had been previously diagnosed.

### 2.3. Computed Tomography

All participants underwent a computed tomography (CT) of the chest and abdomen using a 64-channel multidetector system (Somaton Cardiac Sensations 64, Germany) to quantify total, subcutaneous and visceral abdominal adipose tissue using the equation of Kvist [35]. Visceral abdominal fat (VAT) was determined in a single tomographic cut at L4-L5 intervertebral space level. High VAT was defined as a VAT value above the 75th percentile for sex (122.0 cm^2^ in women and 151.5 cm^2^ in men) [36].

Tomography was also used to assess the coronary artery calcification (CAC) score by the Agatston method [37]. To exclude participants with subclinical atherosclerosis, we only recruited individuals with CAC score = 0.

### 2.4. Assessment of Carotid Intima Media Thickness (CIMT)

The scanning of CIMT was performed with a high-resolution ultrasound equipment in B mode (Sonosite Micromax) and a 13–6 MHz linear transducer. All individuals were evaluated in a supine position with an extended neck. The thickness of the intima and media layers was measured as the distance between the arterial intima-lumen and the media-adventitia regions of the distant wall. At least five determinations were made in the left and right carotid arteries and the CIMT was defined as the average of all measurements. Increased CIMT was considered when the value was above the 75th percentile of the Hispanic population for age and sex [38].

### 2.5. DNA Isolation and Genetic Analysis

Genomic DNA was obtained from peripheral blood leukocytes using conventional kits (QIAamp DNA blood extraction Mini kit, with part number 51106, Qiagen, Hilden, Germany). The quality of samples was corroborated by 260/280 nm absorbance ratio and by 1% agarose gels stained with ethidium bromide. Finally, the DNA concentration was quantified and adjusted to 10 ng/µL using a NanoDrop spectrophotometer (Thermo Fisher Scientific, Waltham, MA, USA). 

A literature review of the *PCSK9* gene polymorphism was performed and we chose the rs2149041polymorphism considering that is located in the promoter region of the gene with a minor allele frequency (MAF) ≥ 5% reported by ALFA (Allele Frequency Aggregator) and 1000 Genomes Project, with previous significant results in disorders where lipid metabolism is associated with cardiovascular diseases. The rs2149041 polymorphism was genotyped using 5′ exonuclease with probes fluorescently labeled with VIC and FAM (TaqMan assay) according to the manufacturer’s instructions (Thermo Fisher Scientific, Waltham, MA, USA). 

The ID for this polymorphic site is ID: C___2018192_10, with sequence: TCATGTGCCCTTTATCTCGAAATTC[C/G]ACTTCCAGGAATTTATGAAACAGAT.

### 2.6. Data Analysis

Statistical analyses were performed using the SPSS software, v24.0. Data are shown as means (standard deviation), percentages, or medians (interquartile range). Regarding data distribution or categorical variables, Student *t*-test, Mann–Whitney U-test, or Chi-square test were performed. The association of the rs2149041 polymorphism was assessed using logistic regression analyses through different genetic models: codominant (*CC* vs. *GG*), dominant (*CC* vs. *CG* + *GG*), and recessive (*CC* + *CG* vs. *GG*). The associated model was adjusted by age, sex, BMI, Cholesterol, and T2DM. Then, the associations of the rs2149041 polymorphism with metabolic variables were adjusted by age, sex, and BMI. Values of *p* < 0.05 were considered statistically significant.

## 3. Results

### 3.1. Study Population

A total of 881 unrelated individuals with a CAC = 0 were included in this study; 732 participants presented CIMT ≤ 75th percentile (CIMT < 75 group) and 149 individuals presented carotid IMT ≥ p75th (CIMT > 75 group) for age and sex [38]. The clinical and metabolic characteristics of the studied groups are depicted in Table 1. 

### 3.2. Association of PCSK9 (rs2149041) Gene Polymorphism with Increased CIMT

The polymorphic site studied was in Hardy–Weinberg equilibrium in the whole sample. The distribution of the rs2149041 polymorphism was similar in both study groups under the codominant and dominant inheritance models. Conversely, the recessive model was associated with an increased risk of developing increased carotid intima media thickness determined by logistic regression with an unadjusted analysis (Model 1, OR = 1.90, 95% IC = 1.16–3.12, *p* = 0.011). Furthermore, the association between the rs2149041 polymorphism and the risk of increased CIMT remained significant with models adjusted by different confounding variables. Model 2: adjusted by age (OR = 1.94, 95% IC = 1.17–3.19, *p* = 0.009); model 3: model 2 + adjusted for sex (OR= 2.10, 95% IC = 1.26–3.46 *p* = 0.004); model 4: model 3 + adjusted for body mass index (OR= 2.11, 95% IC= 1.27–3.50, *p*= 0.004); model 5: model 4 + adjusted for cholesterol (OR= 2.10, 95% IC= 1.27–3.49, *p*= 0.004); and model 6: model 5 + type 2 diabetes mellitus (OR= 2.10, 95% IC= 1.26–3.47, *p*= 0.004), Table 2.

## 4. Discussion

PCSK9 is a protein that participates in the LDL-receptor degradation, so that it is involved in regulating plasma cholesterol levels [39,40,41]. High PCSK9 concentrations in plasma have been associated with vascular disorders [23,24,25,42,43]. Congruently, pharmacological inhibition of PCSK9 with evolucumab reduced the increase in CIMT along the time in patients treated with statin [44] and improved the vascular functionality as determined by carotid stiffness in patients with familial hypercholesterolemia [45]. These observations are in agreement with a rapid reduction of lipid content in carotid plaques achieved with PCSK9 inhibitor alirocumab [46].

In this context, genetic factors related to the PCSK9 expression may be of great relevance in the early assessment of cardiovascular risk, for it has been shown that the genetic component plays a key role in the variability of CIMT [17,20,47]. For these reasons, we decided to evaluate one specific polymorphism in a group of asymptomatic individuals whose cardiovascular risk was estimated by CIMT. Our choice was based on the fact that CIMT has been recognized as a marker or predictor of cerebrovascular events and cardiovascular diseases [47,48,49]. Concerning the polymorphic site (rs2149041) of the *PCSK9* gene, one report has suggested that the presence of the *G* allele may implicate the abolition of a CD28RC element response [50]; congruently, higher LDL-cholesterol reductions were achieved in carriers of the minor allele treated with berberine, a molecule that regulates PCSK9 at the transcriptional level [50,51]. This evidence suggests that the polymorphic site (rs2149041) is a potential genetic factor for evaluating asymptomatic atherosclerosis determined by CIMT. Therefore, we explored the potential link between the *PCSK9* (rs2149041) polymorphism and the susceptibility to present increased CIMT in 881 asymptomatic individuals. Accordingly, a different distribution of the rs2149041 polymorphism was found in both study groups. The association of *GG* homozygote carriers under a recessive model with a risk of increased CIMT, with a ≥ 75th percentile persisted after a logistic regression model adjustment when presenting the most common atherosclerosis triggering factors including age, sex, BMI, cholesterol, and type 2 diabetes mellitus. 

We have to consider that stratification based on CIMT > 75th percentile of age and sex-biased towards increased diabetes and high-cholesterol frequencies in this group since both conditions are statistically associated with CIMT in Hispanic individuals [52] as well as in our study. Therefore, logistic regression was corrected by sex in model 2 (Table 2), by sex and age in model 3, and sex, age, and cholesterol in model 5. In any of these models, *GG* genotype of *PCSK9* rs2149041 gene polymorphism remained significantly associated with high CIMT.

To our knowledge, there are few studies that reported the association of *PCSK9* with CIMT. Norata et al. included 1541 Caucasian individuals and showed an association between the rs505151 polymorphism and increased CIMT [20]. This study further supports that the *PCSK9* genetic background may be of clinical value in CIMT [6]. On the other hand, Ferreira et al. informed that there was a lack of association between rs562556 and CIMT in the STANISLAS cohort [31]. However, our data agree with the report of Norata et al., which suggests the *PCSK9* polymorphisms could be a predictive parameter of clinical events of CAD. Considering that CIMT is a good surrogate of coronary disease [6], our data and one previous report [20] suggest that the *PCSK9* polymorphism could be a predictive parameter of clinical events of CAD. 

Our study has important strengths; for instance, the number of participants well characterized allowed us to assess the association between the *PCSK9* (rs2149041) gene polymorphism and the presence of increased CIMT, adjusted by traditional confounders of cardiovascular risk. Additionally, we evaluated asymptomatic individuals with CAC = 0. Nonetheless, we recognize the main limitation of our study is the sample size in the high-CIMT group and the cross-sectional design of our research. In an opposite situation, the found associations in a large GWAS was carried out with data from the UK Biobank [53], therefore, other studies involving a larger number of participants should be considered. In addition, we analyzed one polymorphic site and therefore, it was not possible to assess linkage disequilibrium. However, we cannot rule out that there are other neighboring regulatory regions in the promoter of this gene that could affect its transcriptional regulation, influencing the risk participation described in this study. Moreover, we recognize that Mexican mestizos constitute an ethnic group with genetic particularities [54]. Consequently, ethnicity is another limitation of the present study since the statistical association found between CIMT and *PCSK9* rs2149041 gene polymorphism may not be extrapolated to other ethnic groups. Further research is needed to determine how different genetic backgrounds modulate the impact of *PCSK9* rs2149041 gene polymorphism over CIMT. Despite this, the findings in the present study suggest its usefulness as a genetic risk marker of increased CIMT in asymptomatic individuals to predict incident atherosclerosis. However, it is important to assess the functional role of this SNP and its clinical plausibility in coronary heart disease progression. In addition, this research provides knowledge in the field of genomic medicine emphasizing clinical and molecular cardiology to performing future panels of genetic markers in the early stages of atherosclerosis that will help prevent or retard its progression and thus avoid later complications.

This research may contribute in the future to integrating data from our population to assess polygenic risk score (PRS). Actually, it would be interesting to carry out a PRS with *PCSK9* variants and CIMT in several populations with distinct background admixture and diverse clinical outcomes, including intermediate phenotypes, which could provide us additional knowledge to link the PRS for cardiovascular diseases and could be implemented in clinical care as a potential marker with an approach of translational risk prediction.

Additional studies, including other *PCSK9* gene polymorphisms, are required in follow-up designs to confirm these findings.

## 5. Conclusions

Our data suggest that the *PCSK9* (rs2149041) polymorphism is associated with an increased risk of increased CIMT in asymptomatic individuals without coronary artery disease determined by the absence of a CAC score. These findings could be useful as markers for the risk of CAD events.

## Figures and Tables

**Table 1:** Clinical and metabolic characteristics of the studied groups.

Characteristics	Carotid IMT ≤ p75 (n = 732)	Carotid IMT ≥ p75 (n = 149)	* *p*
Age (years)	51 ± 9	53 ± 8	0.026 ^¶^
Sex (% male)	40	27	0.003 ^§^
Body mass index (kg/m^2^)	27.6 (25.1–30.6)	28.4 (26.4–31.8)	0.009 ^Ψ^
LDL- cholesterol (mg/dL)	114 (95–132)	121 (101–140)	0.014 ^Ψ^
Non-HDL cholesterol (mg/dL)	140 (120–162)	147 (126–176)	0.012 ^Ψ^
Apolipoprotein B (mg/dL)	91 (75–112)	92 (75–111)	0.920 ^Ψ^
Free fatty acids	0.58 (0.44–0.75)	0.59 (0.47–0.77)	0.428 ^Ψ^
Insulin	17 (12–24)	18 (14–24)	0.314 ^Ψ^
Total cholesterol	189 (165–209)	196 (175–225)	0.001 ^Ψ^
Triglycerides	141 (105–191)	140 (111–208)	0.292 ^Ψ^
HDL-Cholesterol	45 (36–55)	48 (38–57)	0.149 ^Ψ^
LDL-Cholesterol (mg/dL) *	109 (91–127)	118 (98–138)	0.004 ^Ψ^
LDL-cholesterol ≥ 130 mg/dL *	23.0	34.2	0.004 ^§^
Non-HDL cholesterol > 160 mg/dL	26.4	35.6	0.023 ^§^
LDL-cholesterol ≥ 130 mg/dL	27.9	37.6	0.018 ^§^
Apolipoprotein B ≥ 110 mg/dL	26.9	25.5	0.723 ^§^
Visceral abdominal fat > p75	53.5	64.4	0.015 ^§^
Insulin resistance of adipose tissue > p75	48.9	50.7	0.697 ^§^
Type 2 diabetes mellitus	9.3	14.1	0.076 ^§^
Alleles			
C ^§^	66.9	66.8	
G ^§^	33.1	33.2	
Genotypes			
CC ^§^	43.4	50.3	
CG ^§^	46.9	32.9	0.002
GG ^§^	9.6	16.8	

Data are shown as mean ± standard deviation (student *t*-test) ^¶^, percentage (square chi-test) ^§^ or median (interquartile range) (Mann–Whitney U-test) ^Ψ^. These parameters were calculated by Friedewald method *.

**Table 2:** Association of PCSK9 (rs2149041) gene polymorphism with high carotid intima-media thickness.

High Carotid Intima-Media Thickness *p* ≥ 75																		
rs214904 Polymorphism	Model 1: Unadjusted Analysis			Model 2: Adjusted by Age			Modelo 3: Model 2 + Adjusted for Sex			Modelo 4: Model 3 + Adjusted for Body Mass Index			Modelo 5: Model 4 + Adjusted for Cholesterol			Modelo 6: Model 5 + Type 2 Diabetes Mellitus		
	OR 95%	IC	*p*	OR 95%	IC	*p*	OR 95%	IC	*p*	OR 95%	IC	*p*	OR 95%	IC	*p*	OR 95%	IC	*p*
**Codominant**																		
*CC*	Reference																	
*GG*	1.51	0.89–2.55	0.119	1.54	0.91–2.60	0.105	1.69	0.99–2.87	0.053	1.72	1.0–2.93	0.046	1.66	0.97–2.83	0.64	1.71	1.0–2.91	0.049
**Dominant**																		
*CG + GG*	0.75	0.53–1.07	0.124	0.76	0.53–1.08	0.133	0.80	0.56–1.14	0.220	0.81	0.56–1.16	0.260	0.81	0.56–1.16	0.263	0.82	0.57–1.17	0.277
**Recessive**																		
*GG*	1.90	1.16–3.12	0.011	1.94	1.17–3.19	**0.009**	2.10	1.26–3.46	**0.004**	2.11	1.27–3.50	**0.004**	2.10	1.27–3.49	**0.004**	2.10	1.26–3.47	**0.004**

C: dominant inheritance model, the reference group is formed by the major allele homozygote genotype; r: recessive inheritance model, the reference group is formed by the minor allele homozygote genotype. The text in bold denotes statistical significance.

## Data Availability

Data supporting the results are available from the correspondence author upon reasonable request.
